# Why don't hospital staff activate the rapid response system (RRS)? How frequently is it needed and can the process be improved?

**DOI:** 10.1186/1748-5908-6-39

**Published:** 2011-04-16

**Authors:** Stuart D Marshall, Simon Kitto, William Shearer, Stuart J Wilson, Monica A Finnigan, Tamica Sturgess, Tonina Hore, Michael D Buist

**Affiliations:** 1 Southern Health Simulation and Skills Centre, Monash Medical Centre Moorabbin Campus Centre Road, East Bentleigh, Melbourne, Australia; 2 Monash University, Academic Board of Peri-operative Medicine, Commercial Road, Prahran, Melbourne, Australia; 3 Monash University, Department of Surgery, Clayton Road, Clayton, Melbourne, Australia; 4 University of Toronto, Department of Surgery, College Street, Toronto, Canada; 5 Southern Health Quality Unit, Monash Medical Centre Clayton Clayton Road, Clayton, Melbourne, Australia; 6 Monash Medical Centre Intensive Care Unit, Clayton Road, Clayton, Melbourne, Australia; 7 University of Tasmania Rural Clinical School, Brickport Road, Burnie, Tasmania, Australia

## Abstract

**Background:**

The rapid response system (RRS) is a process of accessing help for health professionals when a patient under their care becomes severely ill. Recent studies and meta-analyses show a reduction in cardiac arrests by a one-third in hospitals that have introduced a rapid response team, although the effect on overall hospital mortality is less clear. It has been suggested that the difficulty in establishing the benefit of the RRS has been due to implementation difficulties and a reluctance of clinical staff to call for additional help. This assertion is supported by the observation that patients continue to have poor outcomes in our institution despite an established RRS being available. In many of these cases, the patient is often unstable for many hours or days without help being sought. These poor outcomes are often discovered in an ad hoc fashion, and the real numbers of patients who may benefit from the RRS is currently unknown. This study has been designed to answer three key questions to improve the RRS: estimate the scope of the problem in terms of numbers of patients requiring activation of the RRS; determine cognitive and socio-cultural barriers to calling the Rapid Response Team; and design and implement solutions to address the effectiveness of the RRS.

**Methods:**

The extent of the problem will be addressed by establishing the incidence of patients who meet abnormal physiological criteria, as determined from a point prevalence investigation conducted across four hospitals. Follow-up review will determine if these patients subsequently require intensive care unit or critical care intervention. This study will be grounded in both cognitive and socio-cultural theoretical frameworks. The cognitive model of situation awareness will be used to determine psychological barriers to RRS activation, and socio-cultural models of interprofessional practice will be triangulated to inform further investigation. A multi-modal approach will be taken using reviews of clinical notes, structured interviews, and focus groups. Interventions will be designed using a human factors analysis approach. Ongoing surveillance of adverse outcomes and surveys of the safety climate in the clinical areas piloting the interventions will occur before and after implementation.

## Background

Patients that become critically unwell in a hospital ward environment commonly exhibit a recognisable period of abnormal physiological signs before they suffer a cardiac arrest or other catastrophic event [1-6]. It has been established that early intervention may halt their deterioration and prevent a cardiac arrest or unplanned intensive care unit (ICU) admission. The rapid response system (RRS) is a process whereby health professionals can promptly access help if a patient under their care deteriorates and before they become critically ill to prevent further instability. The type of assistance varies depending on the setting, but typically the medical emergency team (MET) that responds consists of trained specialist staff members such as intensivists and senior nurses.

Many studies [7-9] and a recent meta-analysis [10] showed that the number of cardiac arrests in hospitals can be reduced by the introduction of a RRS. The MERIT study [11], the only multicentre prospective randomised study, initially showed no benefit. A recent *post hoc *analysis of the MERIT data of both intervention and control hospitals, however, demonstrated early intervention using a RRS clearly reduces in-hospital cardiac arrests and mortality [12].

The difficulty in establishing the effectiveness of RRSs and METs has at least in part been due to the failure of clinical staff to call for help early in all circumstances. A review of critical incidents in our own institution, suggested that a failure to call the MET was a common factor in a large proportion of cardiac arrests and unplanned ICU admissions [13]. The reasons behind this failure to call for help have not previously been investigated.

### Failure to activate the RRS

In order to help health professionals to identify when a patient is becoming physiologically unstable, specific criteria based on the vital signs are often fixed for use at the point of care. Deviation outside of these physiological criteria such as those listed in Table 1 represent a state where the patient is thought to be at an increased risk of further deterioration, or has a limited reserve to cope with additional physiological insults [1].

**Table 1:** Medical emergency team call criteria or triggers

Airway	Respiratory Distress Threatened Airway
Breathing	Respiratory Rate > 30 breaths per minute Respiratory Rate < 6 breaths per minute Oxygen Saturation <90% on oxygen

Circulation	Blood Pressure < 90 mmHg despite treatment Pulse Rate > 130 beats per minute

Neurology	Decreased level of consciousness Fitting

Other	Concerned Need of treatment & prompt help

It is currently unclear how many patients in a routine ward environment would meet the abnormal physiological criteria, or if they would progress to an unstable state and benefit from activation of the RRS. Defining the subgroup of patients who would probably have benefitted from early intervention would allow the underlying factors to be more readily investigated and addressed by redesigning of the process and more targeted education for the staff.

Several barriers have already been identified in the literature that prevent the initial implementation of a RRS; failure to view errors as a product on the system rather than individual mistakes, lack of data that METs are life saving, professional control issues, effective education, and financial pressures [14]. It is possible the barriers to ongoing effectiveness are similar, but these have not been identified in the literature. We hypothesise that further barriers exist to prevent the staff members calling for help. These involve both the individual health professionals' internal cognitive processes and cultural expectations from the clinical context and professional identities.

### Theoretical framework

**Figure 1:**
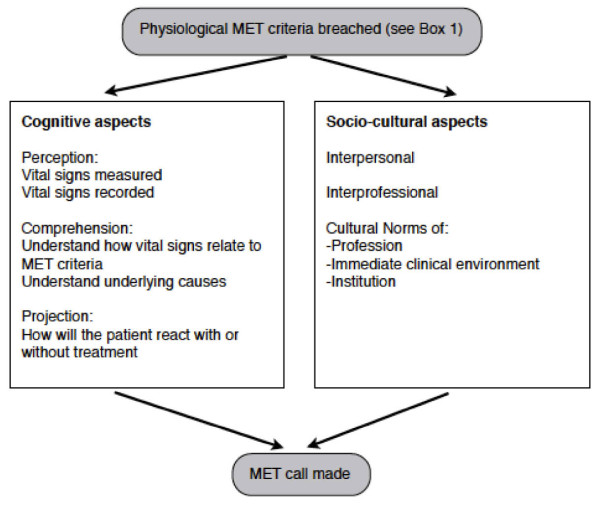
A theoretical framework describing the cognitive and socio-cultural barriers to calling the Medical Emergency Team (MET)

As noted above, no single theory is available to describe why, when patients meet defined criteria, that the staff members do not activate the RRS. We will employ theoretical triangulation [15] using theories from the cognitive engineering model of situation awareness, and sociologically informed models of inter-professional practice [16] to aid further investigation (Figure 1). Both of these theories will be applied in parallel to develop a detailed understanding of the psychosocial process of RRS activation.

### Situation awareness

Situation awareness describes the gathering and understanding of cues in the environment leading to a projection of the possible future consequences [17]. In the circumstance of a deteriorating patient, each health professional makes his or her own assessment of the situation and decides on a resultant course of action, perhaps in consultation with colleagues. The situation awareness model has three distinct parts: perception, comprehension, and projection.

### Perception

Perception of the vital signs of a deteriorating patient typically means that the observations have been taken and transposed to the observation chart. If the patient is deemed to be unwell, these observations will usually be repeated more frequently to ensure that a further deterioration is not occurring and that treatment is effective.

### Comprehension

Comprehension of the meaning of the physiological signs is also important. Specifically with the RRS, the clinical staff must be able to recognise how the patient's observations relate to the triggering criteria. A deeper level of comprehension of the patient's physiological state may occur with more experienced staff members. This may lead to a recollection and comparison to past experiences of similar cases to guide future decision making and information gathering [18]

### Projection of future state

The ability of the health professional to project the future course of events is determined by an understanding of the current state and their previous experiences with similar situations. The rationale given for the clinical staffs' predictions may not always be obvious, even to them. This 'sixth sense' of being able to project the future state is often the result of cues that may not be consciously recognised [19]. One of the advantages of the RRS is that it removes the necessity of the clinical staff to fully understand and diagnose the problem before asking for help. If the health professional looking after the patient is confident they know what the clinical problem is, they may be able to troubleshoot the problem without requiring help. Conversely, if the health professional is junior with only minimal experience, the triggering criteria should trigger them to call for assistance.

### Sociological models of inter-professional practice

Even if the health professional realises the patient fulfils the physiological criteria for activation of the RRS, there may be socio-cultural and political barriers preventing them from calling for help. These barriers may occur between professional groups, within professional groups, or as a result of a group identity existing such as within a ward or specialty area.

### Inter-professional barriers

Barriers may occur at an inter-professional level where there are perceived to be differing levels of trust and cooperation between professional groups [20]. The RRS may be prevented from being activated by levels of distrust between the emergency team attending and the treating groups. Similarly, barriers may be occurring because of the differing views and perceived role of the RRS by nursing and medical staff.

### Intra-professional barriers

Pre-existing pathways to activating the RRS may be based in the culture of the profession [21]. It has been established that nursing staff are more likely to activate the RRS than medical staff [22]. This difference between professional groups could be a result of cultural barriers within the medical profession that have not previously been identified.

### Contextual and local cultural factors

Specific clinical areas of the institution may exhibit differing cultures about the role and function of the MET and pathways to access help. These clinical areas are in turn situated within the complexity of the character of the institution itself. One of the many potential factors that has already been identified in supporting a RRS is whether the hospital has a teaching function [23]. Other local cultural aspects have not been investigated, such as the presence of implicit or explicit directions to seek help from other sources before activating the RRS, which may vary between clinical areas.

The experiences of individuals' interpersonal interactions during MET calls also may have a detrimental effect on future optimal MET call behaviour amongst staff. For example, the attitude of the MET call team on their arrival may have a substantial effect on the culture of the clinical area. If the team is negative and critical, the ward staff may be reluctant to call for help on future occasions, whereas a helpful team that supports and educates the staff will encourage a positive attitude [24].

### Aims of this study

The aims of the proposed study are threefold: to establish the scope of the problem; to examine the barriers to calling the MET; and to pilot a redesign of the RRS to improve its effectiveness.

### Establishing the scope of the problem

First, we intend to determine the prevalence of patients meeting the physiological criteria for activation of the RRS at a number of hospitals. We will identify the number of patients who would have benefited from early intervention but didn't receive it. This will allow a further measure to be developed: the 'missed MET', which will be useful in examining the barriers to calling the MET.

### Examining the barriers to calling the MET

The reasons why the RRS was not activated by ward staff will be determined using the theoretical framework described in figure 1. Health professionals will be approached from all groups involved in RRS activation, from junior and senior medical and nursing staff, to members of the MET themselves to ascertain the common reasons why help may not be called, or called too late. Staff involved in successful and unsuccessful rapid response events will be approached as well as those involved in cases of 'missed MET'.

### Redesigning the MET system

**Figure 2:**
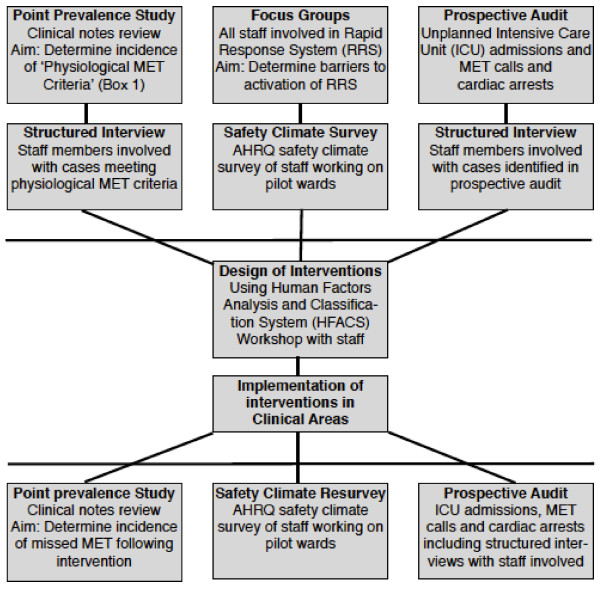
Overview of methods to be used

In any knowledge translation activity, it is essential that the end users of the knowledge are included to ensure that the knowledge and its subsequent implementation are relevant to their needs [25]. Once the scope and barriers to the RRS are understood, we will pilot a redesign of the RRS to increase its effectiveness. Evaluation of these interventions will be determined by repeat measurements from the first two phases of the study (Figure 2).

## Methods

Three related studies will be undertaken concurrently with data collected before and after the design and implementation of an intervention to address the issues identified (Figure 2).

### Point prevalence study

The prevalence of patients meeting the physiological criteria for MET calls will be measured across four hospitals of differing size and caseload over a 24-hour period. These hospitals include an outer suburban 520 bed acute hospital, a small 120 bed elective surgical and oncology centre, a large metropolitan teaching hospital, and an outer suburban community hospital. These four hospitals comprise the majority of the acute care of a health network with over 12,000 staff, 2,100 beds and 180,000 hospital admissions. A team of researchers will examine the clinical notes of all adult in-patients that are not being cared for in critical care areas (ICU, emergency department, or operating theatres). The clinical staff involved with those patients at the time the criteria were attained will be interviewed to determine why a MET call had not been made. Data collected from these structured interviews will be used due to the time constraints of the study and potential to interrupt clinical work. The interview questions will be based on the theoretical framework given in Figure 1. All of the patients identified will be followed up to discharge from hospital to determine if the rate of adverse outcomes in patients meeting the physiological criteria. Particular note will be made to determine if any of these patients subsequently became critically ill, require ICU admission or a cardiac arrest call.

This point prevalence study will be the main study determining the scope of the problem, and to define the 'missed MET' for future study. The data collected from the interviews are expected to be sketchy in terms of determining barriers to the MET call due to the limited time available, but may inform the direction of later phases.

### Focus groups

Knowledge translation activities require an in-depth understanding of the context of the user-groups such as: In what formal or informal structures is the user group embedded? What is the political climate surrounding the user group? To whom is the user group accountable? Are changes expected in any of these? [26]. Therefore, focus group interviews of nursing and medical staff will be used to examine these socio-cultural mediating factors that may influence calling for help using the MET system. A minimum of ten focus group interviews will be taken from representative individuals from the four hospitals using criterion and maximum variation sampling [15]. Participants will be sampled by profession (nursing and medicine) and institution (hospital), and stratified by level of experience within the nursing and medical professions and by institutional location. The participants own experience of the MET call system along with aspects of professional, local, and organisational culture will be sought.

After transcription, themes will be identified from both the focus groups and interviews of barriers to calling of the MET. The Agency for Healthcare Research and Quality (AHRQ) Hospital Survey on Patient Safety Culture (HSOPSC) [27] will be completed by all the clinical staff on the pilot wards before and at three months after the interventions have been introduced. Differences between the responses before and after the intervention on the pilot wards will be analysed using a one-way repeated measures ANOVA.

### Prospective audit

Analysis of all unplanned ICU admissions and cardiac arrests will be performed over an eight-week period before the intervention and an eight-week period three months following implementation of the interventions across all four hospitals in the study.

The clinical notes of all unplanned ICU admissions and cardiac arrests will be examined for evidence of a 'missed MET' in the preceding hours or days. This will determine if an early intervention may have prevented the patient becoming critically ill. Clinical staff involved in the care of a patient that has a 'missed MET' will be interviewed using the same structured interview used in the point prevalence study.

The prospective audit will further allow the barriers to the MET call to be determined from actual cases. Furthermore, the incidence of 'missed MET' will be able to be determined.

### Intervention design

Adapting knowledge to the local context is a crucial component in the knowledge translation process [25]. Up to six common barriers identified from the point prevalence, focus group, interview, and prospective case methods will be determined. These barriers will be presented at a workshop consisting of up to twenty clinical staff involved in the MET call process. Case studies will be used to illustrate how the barriers contribute to 'missed MET' calls, and the participants in the workshop will be asked to provide solutions. The potential solutions will then be categorised using the Human Factors Analysis and Classification System (HFACS), and solutions developed using the Human Factors Intervention Matrix (HFIX) [28]. These potential solutions will be rated in terms of feasibility, acceptability, cost effectiveness, effectiveness, and sustainability. Up to five solutions will be chosen, and be implemented as part of the redesign process. The effects of each individual intervention will not be assessed separately.

Six clinical areas will be chosen from the four hospital sites to introduce the redesigned MET system. These six areas will also have additional point prevalence surveys to determine if the mechanism for dealing with the physiologically unstable patient has changed after introduction of the new system. A further prospective audit will also be used to assess the effectiveness of the redesigned solution.

## Discussion

The care of the deteriorating patient is a priority for most health services because it represents an area of high clinical risk, such that there is a high likelihood of an event occurring with the potential for a poor outcome if a patient becomes critically ill. We hypothesise that an effective MET system will minimise this risk by reducing the occurrence of critical deterioration in ward patients. Timely involvement of specialised clinicians should prevent vital organ system collapse or cardiac arrest. The findings of this study will be important in determining how often and what ways the MET call 'safety net' is used by the junior and senior nursing and medical staff members. In addition, the study will give an insight into why clinical staff fail to call for help when it is needed, and what cognitive or socio-cultural factors are the overriding factors in this. Identification of the barriers to calling for help will hopefully allow the design of effective solutions to bypass them. These solutions may take many forms from technological, to process redesign, financial, education, or policy development for the organization.

It is not clear to what extent this study may be limited by the frequency of poor outcomes that can be directly attributed to a failure to call for help. One of the important aspects of this study will be to examine precisely this rate of occurrence so the phenomenon of failure to act when a patient becomes seriously unwell can be more comprehensively understood.

Ultimately we hope the findings of this study will translate to the implementation of improved systems of care of the deteriorating patient. These in turn will reduce the incidence of unplanned ICU admissions and cardiac arrests and improve the survival of those that do occur through early intervention.

## Competing interests

The authors declare that they have no competing interests.

## Authors' contributions

The design of this study was developed by all of the investigators listed. The project funding was obtained by MF. BS and MB will oversee the conduct of the study. All investigators will be involved in the collection, interpretation, report writing, and dissemination of the results. SM prepared this manuscript for publication with the help of SK and TS, with all of the authors having read and approved the final version of the manuscript.
